# Scrotal calcinosis: a rare clinical image

**DOI:** 10.11604/pamj.2022.42.53.35253

**Published:** 2022-05-18

**Authors:** Anjali Alone, Mayur Bhaskar Wanjari

**Affiliations:** 1 Department of Medical Surgical Nursing, Smt. Radhikabai Meghe Memorial College of Nursing, Datta Meghe Institute of Medical Sciences, Sawangi, Wardha, Maharashtra, India,; 2 Department of Research and Development, Jawaharlal Nehru Medical College, Datta Meghe Institute of Medical Sciences, Sawangi, Wardha, Maharashtra, India

**Keywords:** Scrotal calcinosis, calcium, phosphate metabolism

## Image in medicine

Scrotal calcinosis is an uncommon benign disorder of the scrotal skin characterised by multiple calcified intradermal nodules that occur in the presence of normal calcium and phosphate metabolism. A 32-year-old male comes to the outpatient department with the complaints of progressively increasing multiple painless nodules in the scrotum area in 6 months. He did not have any other medical comorbidities. There was no any discharge from the nodule. On physical examination, multiple nodules on the scrotum and nontender, subcutaneous nodules were studded within the scrotal wall.

**Figure 1:**
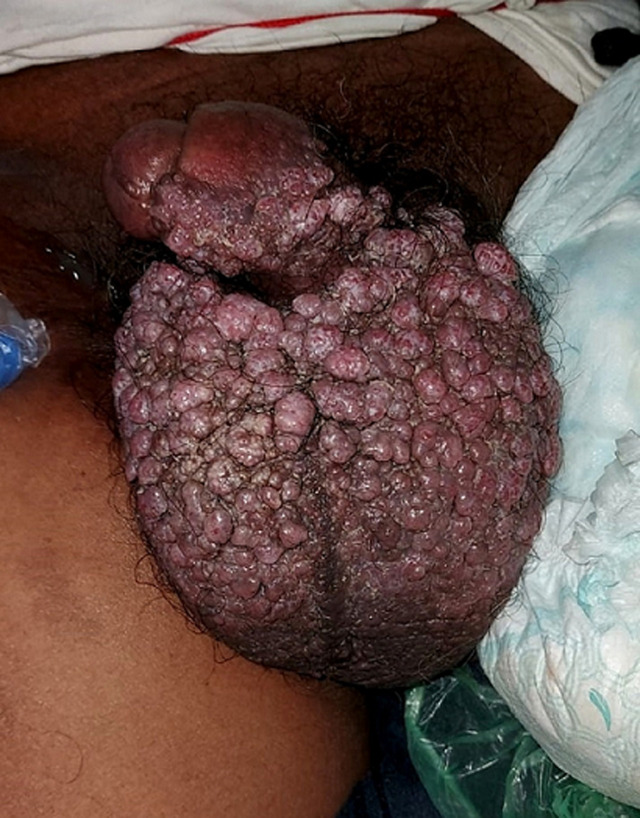
multinodular lesions of the scrotal skin

